# Effect of gonadectomy and hormone replacement on habenula‐induced dopamine inhibition in female and male rats

**DOI:** 10.1111/jne.70183

**Published:** 2026-04-10

**Authors:** Vaughn J. Waldron, Monnette Suttawireesan, Shanzay Fatimah, Istvan J. Merchenthaler, P. Leon Brown

**Affiliations:** ^1^ Maryland Psychiatric Research Center, Department of Psychiatry University of Maryland School of Medicine Baltimore Maryland USA; ^2^ Translational Toxicology, Department of Epidemiology and Public Health University of Maryland School of Medicine Baltimore Maryland USA

**Keywords:** castration, estrogen, sex difference, substantia nigra, testosterone

## Abstract

Sex differences have been noted in the prevalence and severity of several neurological and mental health disorders. Midbrain dopaminergic activity is implicated in the etiology of many of these disorders and therefore may also contribute to some commonly seen sex differences in presentation and treatment. The ability of the lateral habenula to inhibit midbrain dopamine firing activity is reduced in female rats, and we test here the hypothesis that circulating gonadal hormones contribute to this sex difference. In vivo, single unit, extracellular recordings of dopamine neurons were conducted in female and male rats that were intact, gonadectomized, or had hormone replacement. Both spontaneous and habenula‐evoked activities were recorded. In accordance with previous findings, we found that habenular stimulation produces profound inhibition in dopamine neurons that is of longer duration in male rats than female rats. There was no effect of gonadectomy on duration of inhibition in either males or females. Although there was a trend toward stronger rebound excitation in control male rats, there was no significant effect of gonadectomy in either the male or female rats. Here we show that circulating gonadal hormones have no apparent effect on habenular evoked dopamine inhibition. We discuss the limitations of the current study, including the possibility that the influence of circulating gonadal hormones may be limited to sub‐populations of midbrain dopamine neurons.

## 1 INTRODUCTION

It has long been noted that sex‐differences exist in the prevalence and severity of several neurological and mental health disorders. Although substance use disorder is more common amongst men on virtually all other aspects, from escalation, to withdrawal, to treatment, women have a poorer outcome.^1^ Despite a narrowing gap over the past few decades, the prevalence, incidence, and severity of Parkinson's disease are all still greater amongst men than women.^2^ Both major depressive disorder^3^ and anxiety disorders^4^ are about twice as common in women, while the prevalence and severity of schizophrenia are higher in men.^5^ In each of these conditions differences in midbrain dopamine (DA) function, either as a result of neurodevelopment or gonadal hormone milieu, likely plays a contributing role.^6^, ^7^, ^8^, ^9^

While tonic differences in DA function are of interest, so are phasic differences. Midbrain DA neurons of the ventral tegmental area (VTA) and substantia nigra pars compacta (SNc) generally, though not exclusively, are transiently activated by appetitive stimuli and cues^10^, ^11^ and inhibited by aversive stimuli and cues.^12^, ^13^ Activation of the lateral habenula (LHb), via the rostromedial tegmental nucleus (RMTg), provides widespread and powerful inhibition of midbrain DA neurons.^14^, ^15^, ^16^ Such transient inhibition of DA firing is believed to encode negative prediction errors, an important component of associative learning^17^, ^18^; aberrant associative learning is manifested in several of the disorders listed above including depression, addiction, and schizophrenia.^19^, ^20^, ^21^ Given that these disorders display both sex differences and impaired DA‐regulated learning, investigating potential sex differences in the LHb‐RMTg‐DA circuit is warranted.

Previous work demonstrated reduced inhibitory tone exerted by the RMTg on VTA DA neurons in female rats.^22^ We recently expanded upon this finding, showing a similar reduced inhibitory tone exerted by the LHb on SNc DA neurons.^23^ In addition, we showed a higher prevalence of rebound excitation in male rats. Given that both VTA and SNc DA neurons play an important role in several neurological and mental health disorders, and that several of these disorders display sex‐differences in their prevalence, symptoms, and severity, it is reasonable to surmise that sex‐differences in LHb‐evoked DA inhibition may contribute to their presentation. We hypothesized that circulating gonadal hormones alter the ability of the LHb to inhibit midbrain SNc DA neurons and to produce rebound excitation. To test the contribution of sex hormones, we recorded SNc DA neurons in anesthetized male and female rats that were intact, gonadectomized, or gonadectomized with hormonal replacement and determined their response to transient stimulation of the LHb.

## 2 MATERIALS AND METHODS

### 2.1 Animals

Adult Sprague‐Dawley female (200–225 g) and male (225–250 g; both Charles River Laboratories, Wilmington, MA) rats were delivered to the animal facilities at the Maryland Psychiatric Research Center. Rats were single housed upon arrival with ad lib access to food and water. There was a minimum 48‐h acclimation period before any experimental procedures. All brain coordinates were taken from Paxinos and Watson.^24^

### 2.2 Survival surgery

Female rats were randomly assigned to one of three groups based on surgical status: ovariectomized (OVX), ovariectomized with estradiol benzoate (w/EB), or sham controls (Sham). Survival surgery was performed under isoflurane anesthesia delivered via vaporizer (3% in 100% O_2_ to induce, 1%–3% in 100% O_2_ to maintain; MWI, Boisie, ID). Ovariectomy and hormone replacement were done as previously described.^25^ Briefly, a midline incision of the dorsal skin surface was made, followed by an incision of the left oblique muscle to allow access to the peritoneal cavity. The ovary was exteriorized and removed; this procedure was repeated on the right side. In Sham rats, the ovaries were exteriorized but not removed. From the site of the dorsal incision, a silicone‐sealed silicone elastomer capsule (1.47 mm (ID) × 1.96 mm (OD) × 20 mm; Freudenberg Medical, Carpinteria, CA) was inserted subcutaneously at the level of the mid‐scapular region. This capsule was empty for the Sham and OVX group and filled with crystalline estradiol benzoate (Sigma‐Alrich, St. Louis, MO) for the w/EB group.

Male rats were randomly assigned to one of four groups based on surgical status: orchiectomized (ORX), orchiectomized with testosterone (w/T), orchiectomized with dihydrotestosterone (w/DHT), or sham controls (Sham). Under isoflurane anesthesia, orchiectomy and hormone replacement were performed, as previously described.^26^ A midline incision was made along the ventral scrotal surface, followed by an incision of the left cremaster muscle. The testes and testicular fat pad were exteriorized and removed; this procedure was repeated on the right side. In Sham rats, the testes were not removed. A midline dorsal incision was then made, and a silicone‐sealed silicone elastomer capsule (1.47 mm (ID) × 1.96 mm (OD) × 10 mm; Freudenberg Medical, Carpinteria, CA; see Reference [27]) was inserted subcutaneously at the level of the mid‐scapular region. This capsule was empty for the Sham and ORX group and filled with crystalline testosterone or dihydrotestosterone (both Steraloids, Newport, RI) for the w/T or w/DHT groups, respectively.

All incisions were sutured, and the animal was removed from anesthesia and allowed to recover. Rats were given a 3‐day regimen of carprofen (5 mg/kg sc; Norbrook Laboratories, Newry, Northern Ireland) and allowed to recover for at least 14 days before electrophysiological recordings.

### 2.3 In vivo electrophysiology

Rats were anesthetized with urethane (1.5 g/kg ip; Sigma‐Aldrich, St. Louis, MO) and given booster injections as necessary to maintain the plane of anesthesia. A vaginal lavage was performed on female rats. A micropipette with sterile saline (100 μL) was inserted approximately 0.5–1.0 cm into the vagina. Saline was ejected and drawn back up three times, then placed on a glass slide. The fluid was inspected for the relative proportions of leukocytes and epithelial cells (nucleated or cornified). Tissues surrounding the ear canal and the midline of the scalp were treated with 2% mepivacaine (0.15 mL sc; Zoetis Inc., Kalamazoo, MI) and the rat was placed in a stereotaxic instrument (David Kopf Instruments; Tujunga, CA). Body temperature was maintained at 37°C using a feedback‐controlled heating pad. The scalp was incised, and a rectangular section of skull removed to expose the right dorsal brain surface above the LHb and midbrain. The dura was carefully removed for the insertion of stimulating and recording electrodes. A concentric, bipolar electrode (SNEX‐100x, Microprobes, Gaithersburg, MD) was lowered such that the tip was at the ventral portion of the LHb (AP −3.5; ML −1.5; DV −5.1 at 10°). Recording electrodes were prepared from borosilicate glass tubing (1.5 mm (OD) BF‐1401; WPI, Sarasota, FL) using a vertical puller (PE‐2; Narishige; Amityville, NY) and filled with 1 M NaCl. Electrode tips were broken back to produce an in vitro impedance of 5–8 MΩ. Electrodes were attached to a Microdrive (IVM‐1000; Scientifica; Clarksburg, NJ) and lowered through the midbrain SNc (AP −5.4 to −6.2; ML −1.2 to −2.5; DV −6.8 to −8.5) based on stereotaxic coordinates. Individual cells were isolated, electrode potentials were amplified, filtered (0.1–8.0 kHz bandpass), and monitored in real time with an oscilloscope and audio monitor. Electrical activity was digitized at 20 kHz (micro1402; CED; Cambridge, England) and stored for offline analysis (Spike 2; CED; Cambridge, England). Putative DA neurons were identified electrophysiologically.^14^, ^28^ Once isolated, neurons were tested for their evoked response to repeated application of rectangular current pulses to the LHb (biphasic, 1.0 mA, 100 μs, 0.5 Hz) until a minimum of 500 spike events were recorded. Following this, spontaneous activity was observed, again for a minimum of 500 spike events. A maximum of 6 recording tracks were made during the course of an experiment, each separated by 300 μm. For the final recording track, the glass recording electrode was filled with 5% Fast Green in 1 M NaCl and, at the end of recording, was positioned dorsal to the SNc (DV –6.5). Dye was released iontopheretically (−25 μA, 30 min) to later calculate the position of previously recorded neurons. The position of the stimulation electrode was marked with an electrolytic lesion by passing a DC current (−0.1 mA, 8 s) through the electrode. A total of 51 female (15 Sham, 20 OVX, 16 w/EB) and 66 male (18 Sham, 16 ORX, 18 w/T, 14 w/DHT) rats were used for electrophysiology experiments. An average of 2.1 and 3.3 DA neurons were recorded from female and male rats, respectively.

### 2.4 Histology

**FIGURE 1:**
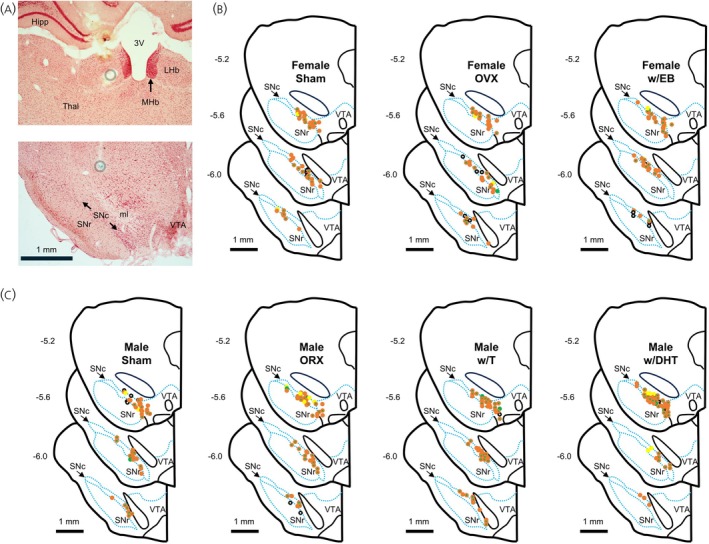
Electrode placement. (A) Representative photos of stimulating (top) and recording (bottom) electrode tracks. Electrolytic lesions (circle) showed placement of stimulating electrodes within the LHb. Recording dye spots (circle) were placed at a fixed point dorsal to the recording area to allow for calculation of recording locations. Anatomical labels are placed for the hippocampus (Hipp), third ventricle (3 V), lateral habenula (LHb), medial habenula (MHb), thalamus (Thal), substantia nigra pars reticulata (SNr), substantia nigra pars compacta (SNc), medial lemniscus (ml), and ventral tegmental area (VTA). Calculated recording locations for each group of female (B) and male (C) rats. Individual cells are marked by their response to LHb stimulation as inhibition (orange), inhibition with rebound (orange with green center), excitation (green) or no change (yellow). Cells from which only spontaneous activity was recorded are marked by a black circle.

Following the recording session, rats were deeply anesthetized (>4.5% isoflurane in 100% O_2_) and, after cessation of respiration, perfused transcardially with 200 mL phosphate buffered saline followed by 500 mL of 10% formalin, both at 4°C. Brains were rapidly removed and immersed in 10% formalin overnight. Brains were then equilibrated to a solution of 30% sucrose in PBS, frozen, and sectioned in the coronal plane at 40 μm on a cryostat (CM 3050S; Leica; Deer Park, IL) through the LHb and SNc. Sections were collected in saline, slide mounted, and counterstained with 0.1% neutral red for contrast. Lesion and dye locations were determined by visual inspection (BX41; Olympus; Center Valley, PA) and recording locations were calculated based on Paxinos and Watson.^24^ Evoked responses were retained for analysis only if the stimulating probe was placed withing the boundaries of the LHb. Both evoked and spontaneous activity were retained only if the recorded neuron was calculated to be within the boundaries of a dopaminergic area (SNc or lateral VTA). Neurons ≤1 mm lateral of the midline (i.e., central and medial VTA) were excluded from analysis (Figure 1).

### 2.5 Data analysis

Evoked and spontaneous firing properties were determined in a manner similar to previously used procedures.^23^ Parameters obtained from spontaneous activity during baseline recording included firing rate, interspike interval (ISI), coefficient of variation of the interspike interval (CV), percent of spikes in burst, and average spikes per burst. Bursting parameters were calculated for all neurons by analyzing bouts of burst firing (initial spike pair with an ISI ≤80 ms and terminal spike pair with an ISI ≥160 ms^29^). Firing patterns were analyzed using autocorrelograms with a 2 s time window and 5 ms bin widths obtained from spontaneous activity during baseline recording.^30^ Neurons were classified as burst firing only if their autocorrelogram displayed a rapid rise with a peak in the first 100 ms followed by a trough and return to steady state, and exhibited a minimum of three, three‐spike bursts over the course of 500 spikes. Neurons exhibiting three or more equally spaced autocorrelogram peaks occurring at multiples of the mean ISI were classified as pacemaker. All other neurons were classified as irregular.

Peri‐stimulus time histograms (PSTH) of spike events were constructed from 0.5 s before to 1.5 s after LHb stimulation using 1 ms bin widths. Cumulative summation plots were constructed from PSTHs by adding the contents of each bin to a running sum of all previous events.^16^ Baseline activity was established with the slope of a least squares regression line fit to the 0.5 s of prestimulus data. Likewise, a least squares regression line was fit to the post‐stimulus activity and neurons were classified as displaying inhibition, excitation (>30% decrease or increase in slope, respectively), or no change in response to LHb stimulation. The duration of the evoked response and the firing rate during the evoked response were also calculated. Rebound excitation following an initial inhibition was also determined by a >30% increase in slope above baseline activity. For each group, a mean PSTH was calculated from all neurons regardless of response using a 25‐point exponential weighted moving average.^14^, ^23^ Data were analyzed in a blind manner using Sigmaplot 12.0. Categorical data were analyzed using chi‐square tests; ordinal data were analyzed with Mann–Whitney U or Kruskall–Wallis tests with post‐hoc Dunn test. All other tests were analyzed by *t*‐test or ANOVA with post‐hoc Tukey tests.

## 3 RESULTS

### 3.1 Gonadectomy and hormone replacement alter weight gain in both sexes

**FIGURE 2:**
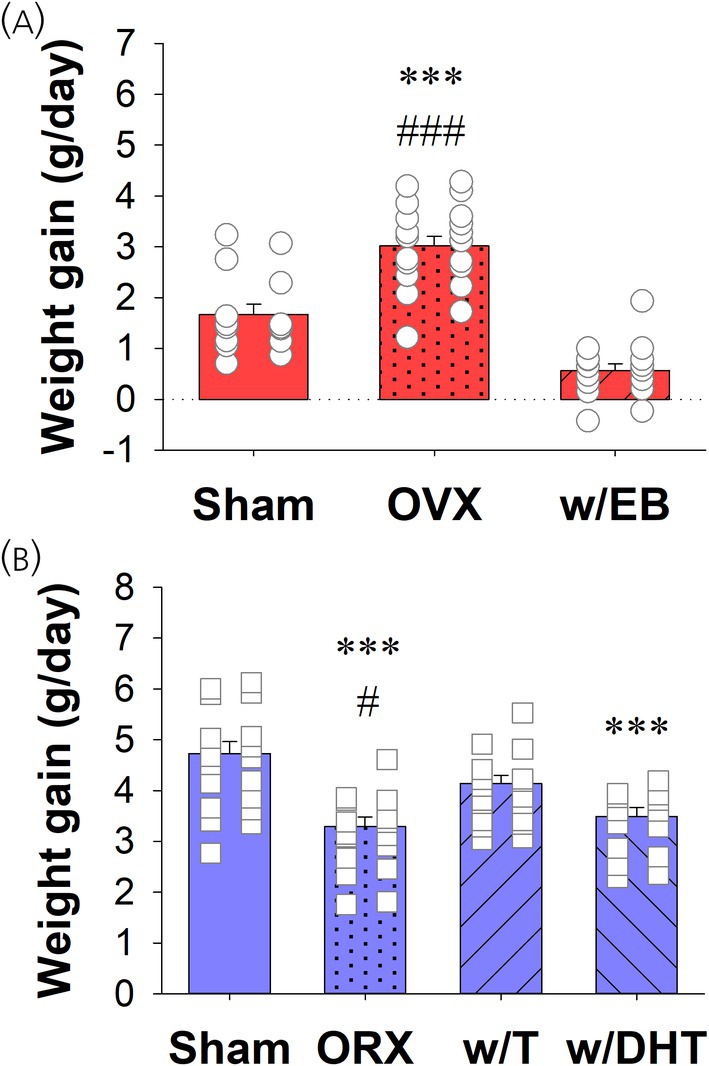
Average daily weight gain between surgical and recording day. Bar charts represent the mean, SEM, and individual data points for each group. (A) In female rats, OVX rats had significantly greater weight gain than Sham (***, *p* <.001) or w/EB rats (###, *p*<.001). (B) In male rats, ORX rats had significantly less weight gain than Sham (***, *p*<.001) or w/T (#, *p* <.05) rats. w/DHT rats also had less weight gain than Sham rats (***, *p* <.001).

Vaginal lavage showed that OVX rats had predominately leukocytes, while w/EB rats had predominately cornified epithelial cells, which is consistent with previous work on the effects of gonadectomy and hormone replacement in female rats.^31^ A comparison of weight change between surgery day and recording day showed a significant group difference in average daily weight change (F_2,48_ = 49.63, *p* <.001; Figure 2A), with OVX females having more weight gain than Sham and w/EB rats (Tukey, *p* <.001), which is also consistent with previous findings of weight gain following ovariectomy and its reversal by estradiol in female rats.^32^

In male rats there was a significant group difference in average daily weight change (F_3,62_ = 11.29, *p* <.001; Figure 2B), with Sham males having more weight gain than ORX and w/DHT rats (Tukey, *p* <.001). In addition, w/T males had more weight gain than ORX rats (Tukey, *p* = .02) without being significantly different from Sham rats. This is consistent with previous findings of reduced weight gain following orchiectomy and its reversal by testosterone, but not dihydrotestosterone, in male rats.^33^

### 3.2 Spontaneous activity in DA neurons unaltered by gonadectomy and hormone replacement in both female and male rats

**FIGURE 3:**
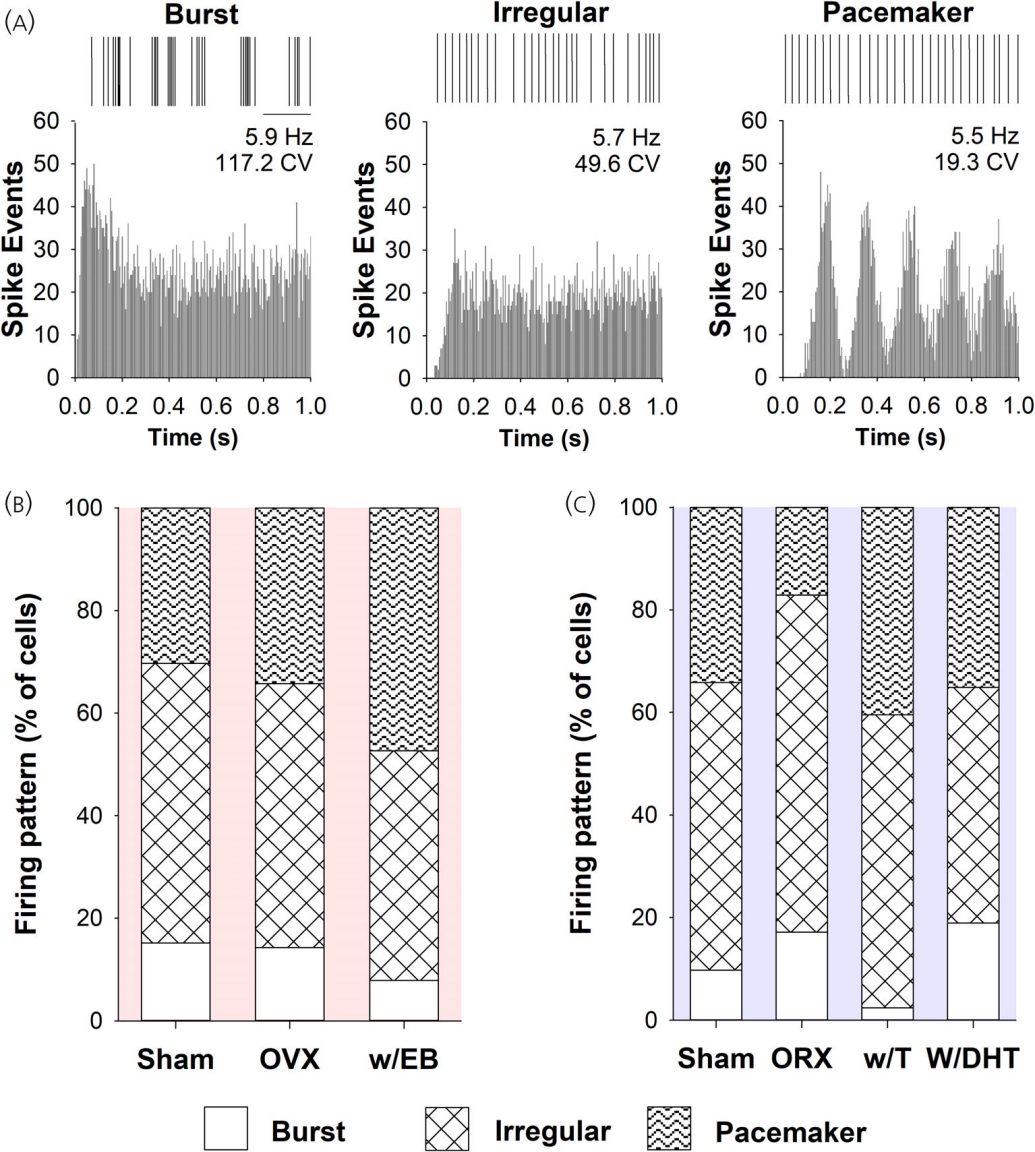
Analysis of firing pattern in SNc DA neurons. (A) Recorded neurons displayed one of three firing patterns: bouts of spikes followed by long pauses (burst), variable pauses between spikes (irregular), or consistent pauses between spikes (pacemaker). Five second representative samples of spike train event markers from three separate neurons are shown, one for each firing pattern (top row, scale bar = 1 s). Corresponding autocorrelograms and CVs for each neuron are profoundly different for each firing type despite similar firing rates (bottom row, see methods for details). Histograms displaying the proportion of each firing type for female (B) and male (C) rats by group. There were no significant differences by group within each sex (see results for details).

In female rats a total of 165 DA neurons were recorded; spontaneous activity was collected from 108 of these neurons (33 Sham, 37 OVX, 38 w/EB) and data are presented in Table 1. Groups did not differ on any spontaneous measure including firing rate (F_2,105_ = 0.08, *p* = .92), coefficient of variation of the interspike interval (CV of ISI; H_2_ = 4.36, *p* = .11), percent of spikes in burst (H_2_ = 2.88, *p* = .24), and mean spikes per burst (H_2_ = 1.74, *p* = .42). Autocorrelograms (Figure 3A) were used to determine firing pattern, which did not differ by group (Chi‐square_4_ = 2.82, *p* = .59; Figure 3B).

**TABLE 1:** Spontaneous and evoked activity in SNc DA neurons from female rats.

Group	Sham	OVX	w/EB
	Spontaneous		
Firing rate, Hz (SEM)	4.79 (0.29)	4.87 (0.25)	4.73 (0.25)
CV of ISI, median (IQR)	32.42 (25.51–53.63)	37.18 (29.43–55.76)	30.86 (22.02–47.98)
% SIB, median (IQR)	2.10 (0.00–20.21)	3.73 (0.59–14.56)	1.12 (0.00–11.20)
Spikes/burst, median (IQR)	2.47 (2.00–4.47)	2.58 (2.00–3.40)	2.29 (2.00–2.70)
	Evoked		
Duration of inhibition, ms (IQR)	48.00 (37.00–67.50)	51.00 (39.50–76.00)	54.00 (35.00–78.00)
Duration of rebound, ms (IQR)	102.00 (72.50–131.50)	118.00 (76.5–162.00)	94.00 (63.5–136.00)
Slope of max firing (Hz/s) (IQR)	60.43 (38.71–106.19)	82.99 (42.25–123.85)	59.71 (41.05–97.23)

In male rats a total of 218 DA neurons were recorded; spontaneous activity was collected from 155 of these neurons (41 Sham, 35 ORX, 42 w/T, 37 w/DHT) and data are presented in Table 2. Groups did not differ on most spontaneous measures including firing rate (F_3,151_ = 0.11, *p* = .95), CV of ISI (H_3_ = 4.25, *p* = .24), and percent of spikes in burst (H_3_ = 5.07, *p* = .17). There was a significant overall difference in spikes per burst (H_3_ = 8.70, *p* = .03) with ORX rats having more than w/T rats (Dunn's method, *p* <.05). Similarly, there was a trend toward reduced pacemaker activity in ORX and reduced bursting activity in w/T rats, but firing pattern as determined from autocorrelograms did not differ by group (Chi‐square_6_ = 10.59, *p* = .10; Figure 3C). This is in keeping with previous research on the effect of gonadectomy on spontaneous SNc DA neurons in male rats.^34^ With the exception of spikes/burst in males, gonadectomy with or without hormone replacement had no effect on spontaneous activity of dopamine neurons in either sex.

**TABLE 2:** Spontaneous and evoked activity in SNc DA neurons from male rats.

Group	Sham	ORX	w/T	w/DHT
	Spontaneous			
Firing rate, Hz (SEM)	4.56 (0.21)	4.40 (0.28)	4.40 (0.21)	4.39 (0.30)
CV of ISI, median (IQR)	32.08 (23.28–51.72)	36.22 (29.51–46.28)	31.78 (23.25–44.64)	37.58 (26.15–48.05)
% SIB, median (IQR)	2.63 (0.11–14.40)	5.63 (0.36–22.45)	1.37 (0.00–7.37)	2.81 (0.22–20.75)
Spikes/burst, median (IQR)	2.29 (2.08–2.55)	2.85^a^ (2.00–3.07)	2.03 (2.00–2.52)	2.25 (2.00–2.79)
	Evoked			
Duration of inhibition, ms (IQR)	61.00 (43.00–78.25)	60.00 (41.00–75.00)	54.50 (42.75–83.00)	54.00 (44.25–66.25)
Duration of rebound, ms (IQR)	89.50 (65.00–133.00)	105.00 (83.00–140.25)	117.00 (89.75–170.75)	97.50 (67.00–126.00)
Slope of max firing (Hz/s) (IQR)	87.65 (46.85–117.86)	55.17 (29.37–101.75)	70.40 (34.15–124.73)	64.21 (35.63–104.99)

^a^ *p* <.05 (compared to w/T group).

### 3.3 LHb evoked inhibition of DA neurons unaltered by gonadectomy and hormone replacement in both female and male rats

**FIGURE 4:**
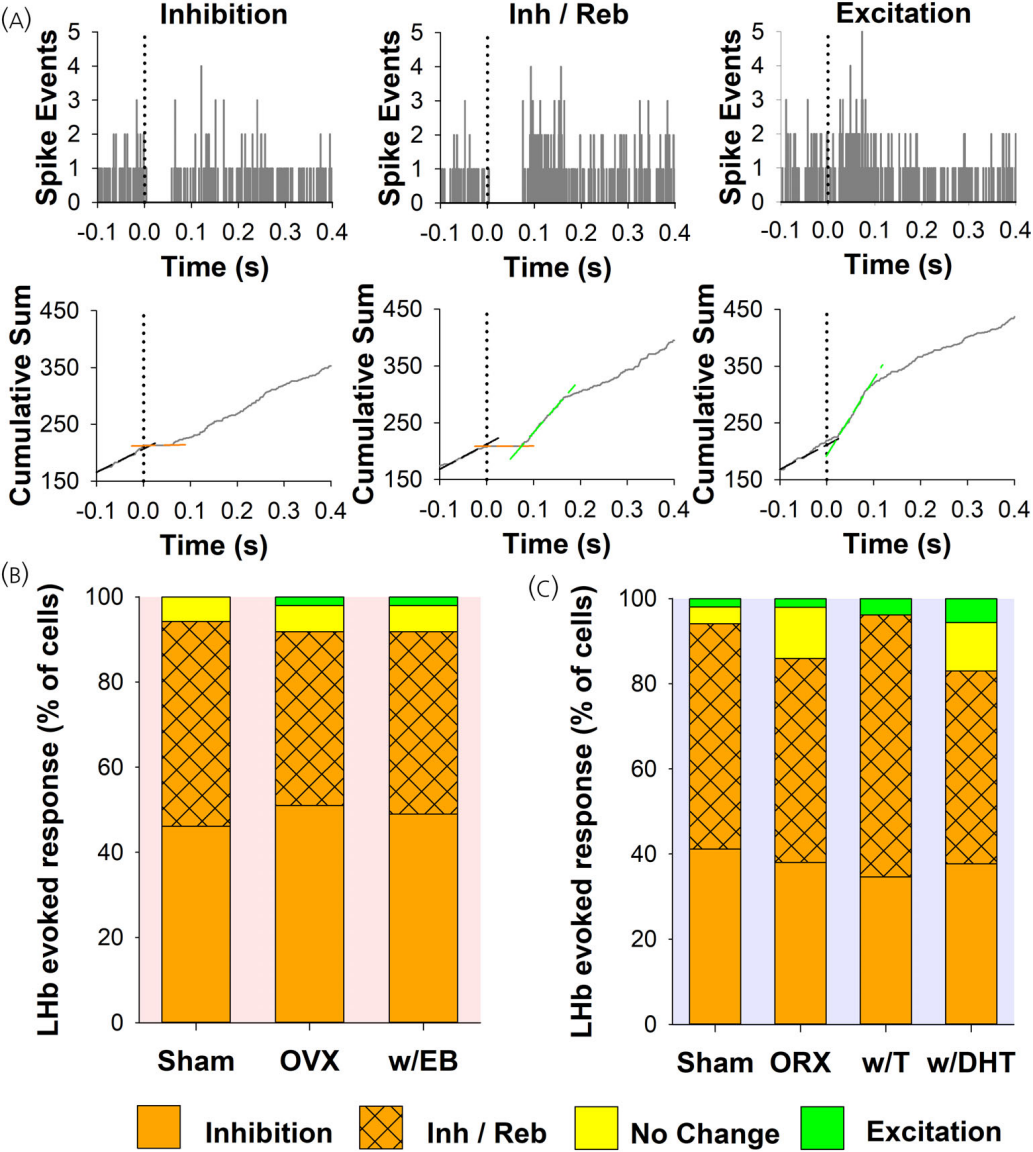
Analysis of LHb‐evoked response in SNc DA neurons. (A) Recorded neurons displayed one of four firing patterns in response to LHb stimulation: inhibition, inhibition with rebound, excitation, or no change. Representative PSTH samples from three separate neurons with evoked responses are shown (top row). Corresponding cumulative summation line graphs clearly demonstrate evoked responses as measured by changes in slope (bottom row, see methods for details). Histograms displaying the proportion of each response type for female (B) and male (C) rats by group. There were no significant differences by group within each sex (see results for details).

DA neuronal firing in response to LHb stimulation was obtained from 150 neurons in female rats (52 Sham, 49 OVX, 49 w/EB). PSTH and cumulative summation graphs (Figure 4A) were used to determine evoked changes in firing rate. The predominant initial response was inhibition in all groups, with a minority of cells exhibiting excitation or no change, and with no significant difference in response prevalence among groups (Chi‐square_4_ = 1.09, *p* = .90; Figure 4B). Amongst neurons that were initially inhibited there was no group difference in the prevalence of rebound excitation (Chi‐square_2_ = 0.43, *p* = .81) and groups did not differ in duration of inhibition (H_2_ = 0.97, *p* = .62) nor duration of rebound excitation (H_2_ = 2.13, *p* = .35; Table 1).

DA neuronal firing in response to LHb stimulation was obtained from 206 neurons in male rats (51 Sham, 50 ORX, 52 w/T, 53 w/DHT). As with female rats, the predominant initial response to LHb stimulation was inhibition in all groups. While there was a trend toward greater prevalence of inhibition in the Sham and w/T groups, this did not reach significance (Chi‐square_6_ = 9.87, *p* = .13). Amongst inhibited neurons, the prevalence of rebound excitation did not differ by group (Chi‐square_3_ = 1.10, *p* = .78; Figure 4C). No significant differences between groups were found in duration of inhibition (H_3_ = 1.07, *p* = .79) or duration of rebound excitation (H_3_ = 5.54, *p* = .14; Table 2).

### 3.4 Overall effect of LHb stimulation on firing activity of DA neuronal population

**FIGURE 5:**
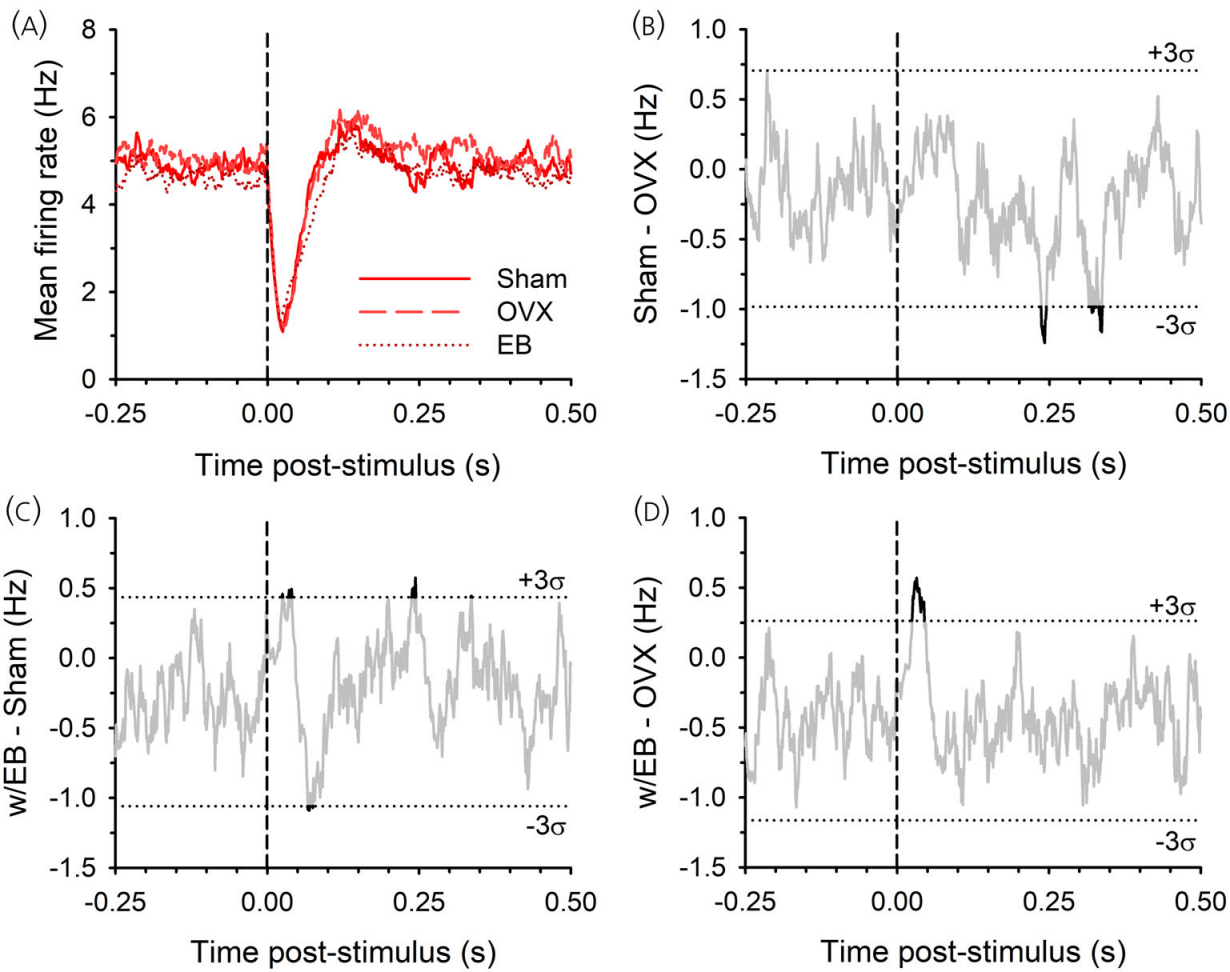
Mean PSTH for all recorded neurons from female rats (A) shows a large degree of overlap among groups. The difference in firing rates is shown between Sham and OVX rats (B), Sham and w/EB rats (C), and w/EB and OVX rats (D).

To capture the overall effect of LHb stimulation on the population of DA neurons, including those neurons with no response or excitation, a mean PSTH of all recorded neurons for each of the groups was constructed using an exponential weighted moving average.^14^, ^23^ In female rats there was a clear, population‐wide inhibition of DA neurons following LHb stimulation in all three groups (Figure 5A). Line graphs of group differences in mean firing rate were also constructed. There was essentially no effect of ovariectomy (Figure 5B) or of estrogen replacement (Figure 5C); differences between the Sham and OVX groups (Figure 5B) and the Sham and w/EB groups (Figure 5C) stayed within three standard deviations of the baseline mean group difference except for some brief periods (<10 ms). The difference between w/EB and OVX groups (Figure 5D) exceeded three standard deviations from 24 to 45 ms after LHb stimulation, representing a slightly weaker initial inhibition in the w/EB compared to the OVX group (Figure 5D).

**FIGURE 6:**
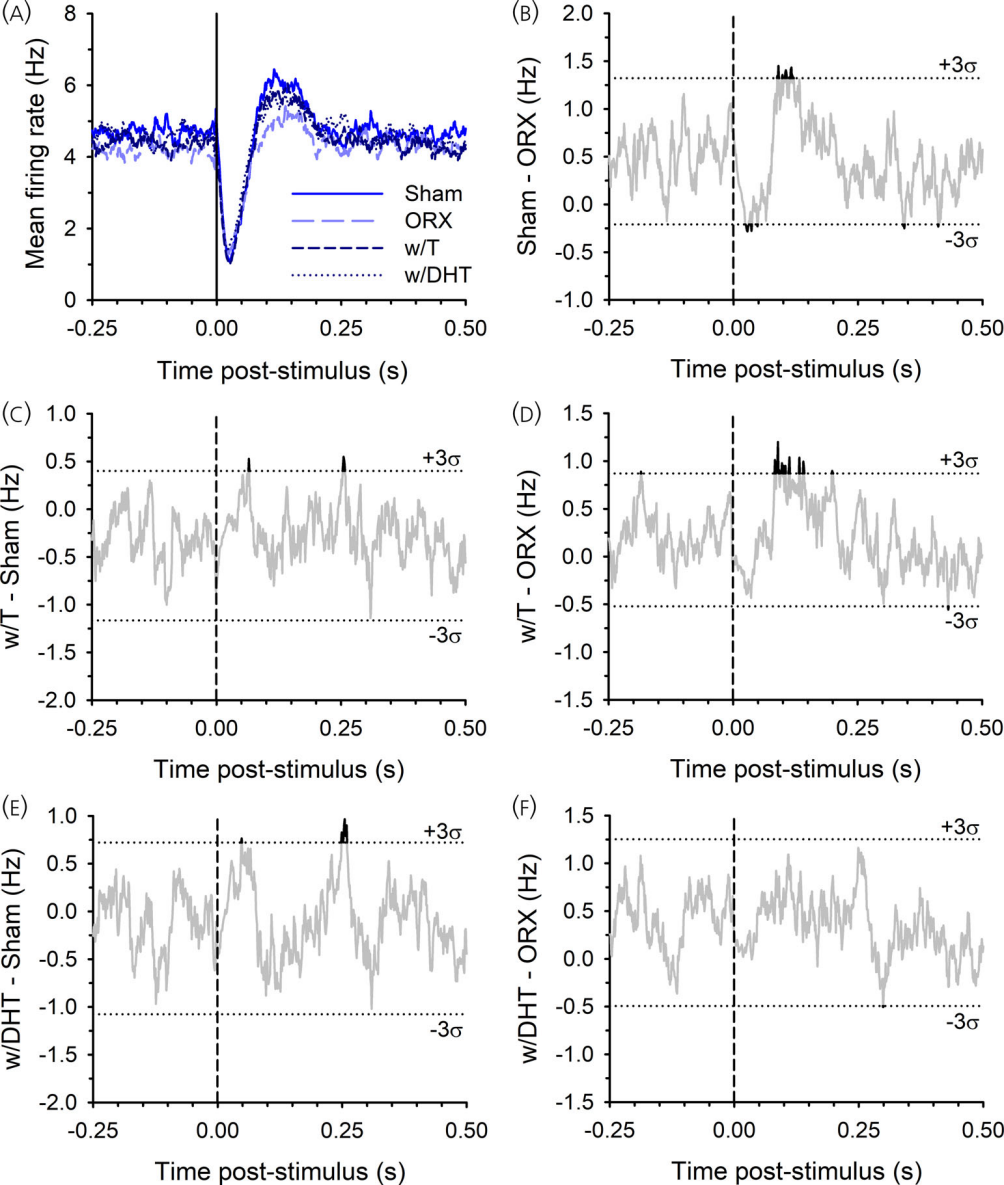
Mean PSTH for all recorded neurons from male rats (A) and the differences between the different groups (B)–(F). There was a trend toward stronger rebound in the Sham male rats, but this difference was not significant (see text for details).

**FIGURE 7:**
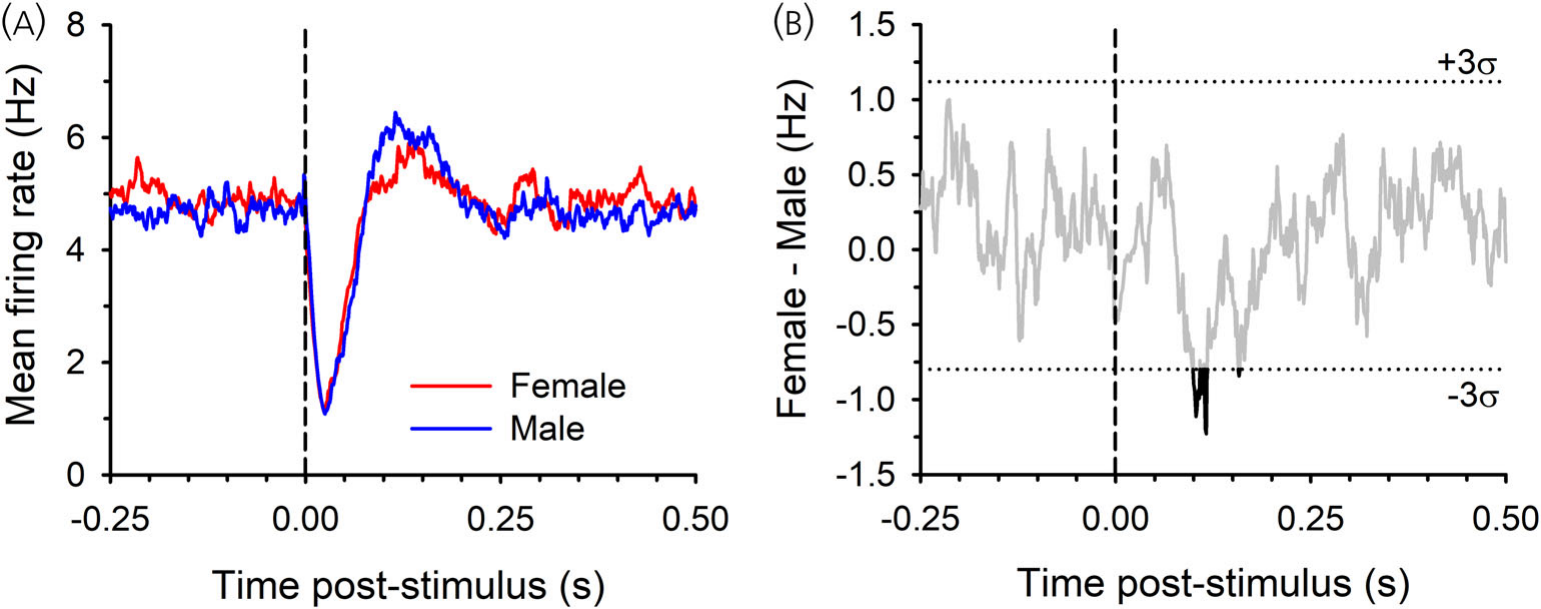
Mean PSTH for all recorded neurons from Sham operated female and male rats (A) and their difference in firing rate (B).

In male rats, there was also a clear population‐wide LHb‐evoked initial inhibition of DA neurons (Figure 6A). A direct comparison between Sham and ORX groups (Figure 6B), where the difference in inhibition exceeded three standard deviations at several points between 24 and 50 ms after LHb activation, indicated a slightly weaker inhibition in ORX rats. This was not recapitulated in the w/T or w/DHT group, with these groups having only slightly weaker inhibition compared to Sham exceeding three standard deviations for some brief periods (<10 ms; Figure 6C,E), and which did not differ from ORX rats (Figure 6D,F). Rebound excitation was prevalent in the Sham males and slightly reduced in ORX males, with the difference exceeding three standard deviations at several points from 88 to 122 ms after LHb activation (Figure 6B). This rebound was also pronounced in the w/T group, with a larger difference compared to the ORX group at several points from 84 to 144 ms (Figure 6D), but less pronounced in the w/DHT group (Figure 6E). We also compared the mean PSTH of Sham female and male rats (Figure 7A). While both groups showed a clear inhibition, the male rats showed a more pronounced rebound excitation. Difference scores showed a group difference in the rebound that exceeded three standard deviations from 99 to 118 ms after LHb activation (Figure 7B).

**FIGURE 8:**
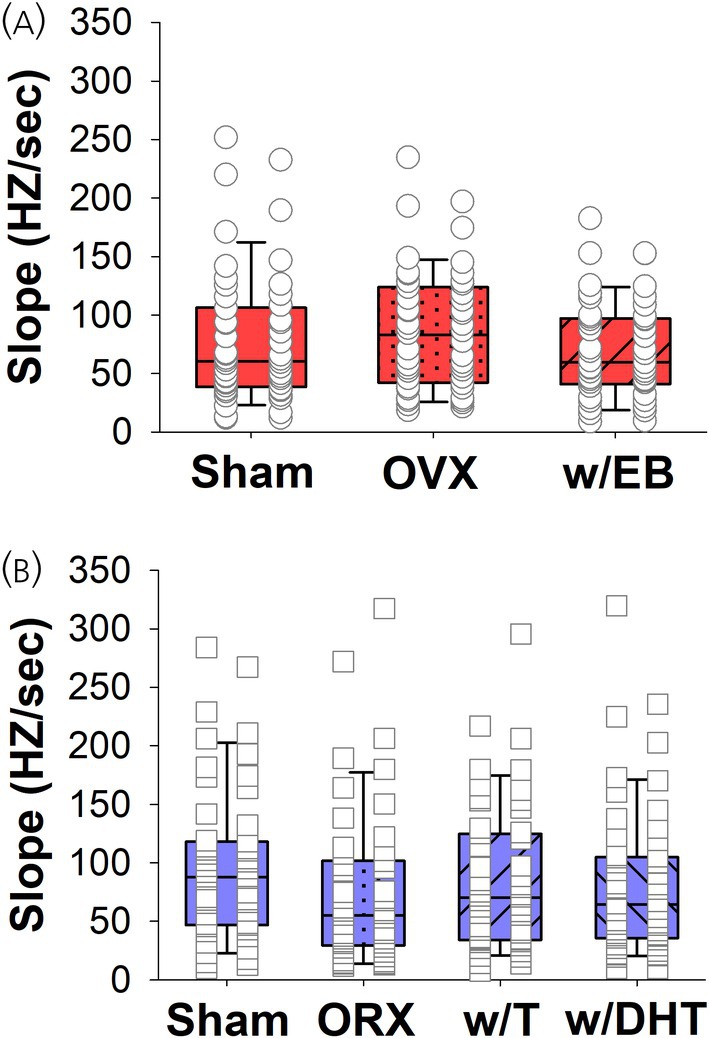
Box plots with the maximal change in firing rate as measured by slope. Boxes represent the median and IQR, whiskers represent the 10%–90% range, and individual data points are overlaid. There were no significant differences between groups in either (A) female rats or (B) male rats, nor was there a significant difference between Sham female and male rats (see text for statistical details).

**FIGURE 9:**
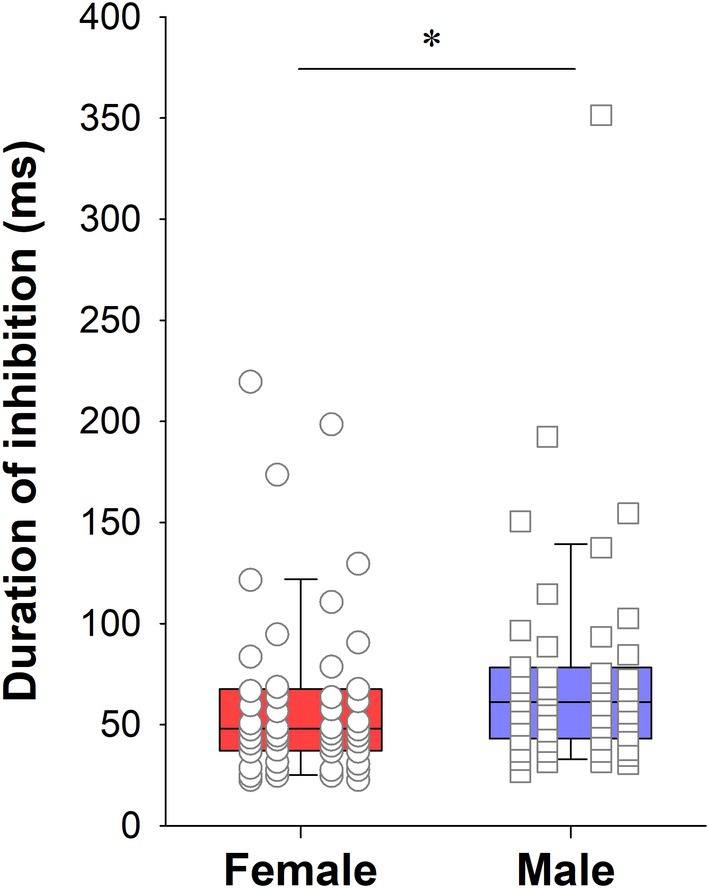
Box plot with duration of inhibition for Sham operated female and male rats, which was significantly longer in male rats (*, *p*<.05). Boxes represent the median and IQR, whiskers represent the 10%–90% range, and individual data points are overlayed.

To better quantify the strength of the rebound excitation, we calculated the slope between the minimum firing rate (from 0 to 100 ms post stimulation) and maximum firing rate (from 100 to 300 ms post stimulation) for each individual neuron, with a steeper slope indicating a more pronounced rebound. This analysis confirmed there were no significant differences amongst the female groups (H_2_ = 1.93, *p* = .38; Table 1; Figure 8A). While the findings in male rats were generally consistent with what was shown in Figure 6, with the Sham and w/T groups having a stronger rebound, there were no significant differences amongst the male groups (H_3_ = 6.02, *p* = .11; Table 2; Figure 8B). Though male rats displayed steeper slopes, the difference between the Sham groups for each sex was not significant (Mann–Whitney U = 1054, *p* = .07). Finally, while there was no apparent difference in the initial inhibition, we did find that the duration of inhibition was longer in males than females (Mann–Whitney U = 899, *p* <.05; Figure 9).

## 4 DISCUSSION

### 4.1 Main findings

Although previous work has shown spontaneous DA activity varies with the estrous cycle,^35^ an effect which can be blocked by ovariectomy and recovered with hormone replacement,^36^ we did not see a similar effect here. This is potentially due to the fact that those studies recorded DA neurons from the VTA, while we recorded almost exclusively from the SNc. Work on the effect of estrogen on the nigrostriatal pathway has shown changes in behavior, such as amphetamine‐induced rotation,^37^, ^38^ downstream DA release,^37^, ^39^ DA neuronal density,^40^ and DA synthesis.^41^ However, there is a lack of electrophysiological work on the effect of estrogen on SNc DA neurons. Early work showed a mixture of increases and decreases in SNc DA firing rate following iv estrogen,^42^, ^43^ a result also seen with iontophoretic application.^44^ Although estrogen receptor beta has been demonstrated in the SNc, it is largely in the lateral portion^45^; consequently, estrogen effects on SNc DA neurons may be limited to that specific subregion. Previous work has shown no effect of orchiectomy on SNc DA cell spontaneous firing, although orchiectomy does increase VTA DA burst firing in a testosterone‐sensitive manner.^34^ Our data largely comport with these previous findings.

We found no overall effect of ovariectomy or orchiectomy on LHb‐evoked activity when comparing individual neurons, although we did see a difference in the duration of inhibition between Sham females and males, consistent with previous findings.^22^, ^23^ Since subtle differences in prevalence, strength, and duration of evoked activity as well as subthreshold effects may contribute to the population level effect, we analyzed the summed response of neurons regardless of response type. In this analysis, we found no effect of ovariectomy or orchiectomy on LHb‐evoked activity, although there was a trend toward smaller rebound excitation in the ORX rats and the Sham female rats compared to Sham male rats. It is of note that previously we found a small, but significantly stronger rebound excitation in male rats compared to female rats.^23^ Our purpose for including the w/DHT group was to test whether any potential difference found with testosterone was driven by aromatization to estrogen. Although there was a trend toward reduced strength of the rebound excitation in the w/DHT group, there were no significant differences between male groups rendering this comparison moot. It is possible that the rebound phenotype is limited to a subgroup of SNc DA neurons and was diluted by the broad sampling from the SNc done here.

Previous work has shown that following stimulation of inhibitory pathways a majority of midbrain DA neurons show rebound excitation that is intensity dependent,^46^ a phenotype which is also seen in anesthetized animals with noxious foot shock^47^ and in awake animals with aversive environmental stimuli.^48^ Further work has shown that within the ventral SNc there are DA cells that appear to be intrinsically ‘rebound ready’,^49^, ^50^ similar to the rebound excitation we report here. Such rebound excitation has been hypothesized to signal relief following the termination of an undesirable stimulus in an opponent‐process like fashion^51^; to motivate an organism to escape from an aversive situation^48^; or to act as a safety signal that confirms the termination of a stressor,^52^ potentially reducing anxiety associated with stressors. Indeed, in the Vogel conflict test, a measure of anxiety resulting from co‐administered rewards and punishers, male rats accept more punished rewards than females,^53^ as do male rats administered high levels of testosterone^54^ and female rats given a hypothalamic administration of an anabolic steroid.^55^ If it is the case that such a relief or safety signal is regulated by the presence of testosterone, then that may provide a mechanism to explain known sex differences in anxiety^56^, ^57^ and impulsivity.^58^

### 4.2 Limitations

While we have shown a longer duration of inhibition in male rats than female rats here, the effect was not as strong as previously shown.^22^, ^23^ This may result from the fact that the female and male groups were not run concurrently in the present study, but sequentially. Small changes in procedure, such as rotation of lab members performing procedures or seasonal changes in animal husbandry, may contribute to such an outcome. It is worth noting that all of the male groups had median durations of equal or longer duration than all of the female groups, regardless of hormonal status. This would suggest that the sex difference in duration of LHb‐induced inhibition results from differences in earlier neurodevelopment.

Since a combination of subtle differences in prevalence, strength, and sub‐threshold rebound may combine to have a population level effect that is not detectable with individual neuronal analysis, we looked at the summed effect of all neurons regardless of evoked response. Though there were trends in the data, such as a stronger rebound in Sham male rats compared to Sham female and ORX rats, these differences were not significant. It is possible that there is a difference between these groups but that the effect is subtle and requires a larger sample, or that the effect is limited to a subpopulation of SNc DA neurons. We will note that recent work showed DA neurons projecting to the dorosolateral striatum have a biophysical profile that allows for rapid rebound bursting in response to hyperpolarization^59^; the researchers, however, did not record from female mice. A targeted analysis of just these DA neurons may reveal greater group differences in rebound excitation than seen here with our broader sampling approach.

It should be noted that we only analyzed neurons greater than 1 mm from the midline, meaning these data come almost exclusively from SNc DA neurons. As mentioned previously, there is a relative paucity of research on SNc DA neurons compared to VTA DA neurons, despite the fact that the SNc is of importance not only in Parkinson's disease^60^ but is also implicated in addiction,^61^ schizophrenia,^62^ and other neuropsychiatric disorders.^63^ However, given the known heterogeneity of basal activity, inputs to and projections from midbrain DA areas, the results seen here may not apply to VTA DA neurons.

### 4.3 Conclusions

Sex differences in the prevalence and severity of several neuropsychological ailments, and the biological factors that may underlie such differences, are of continuing research interest. The present study demonstrates how DA activity, which is implicated in several of these ailments, may be regulated by sex and gonadal hormones. Further work on these regulating factors has the potential to shed light on the etiology and treatment of several mental health disorders.

## AUTHOR CONTRIBUTIONS

**VJW**: Data curation, formal analysis, investigation, methodology, writing—review and editing, visualization. **MS**: Formal analysis, investigation, writing—review and editing, visualization. **SF**: Formal analysis, investigation, writing—review and editing, visualization. **IJM**: Conceptualization, methodology, writing—review and editing, visualization. **PLB**: Conceptualization, data curation, formal analysis, funding acquisition, investigation, methodology, project administration, resources, supervision, writing—original draft, writing—review and editing, visualization.

## CONFLICT OF INTEREST STATEMENT

The authors have no conflicts of interest to declare.

## ETHICS STATEMENT

This study was conducted in strict accordance with The Guide for the Care and Use of Laboratory Animals (National Research Council, 2011). All procedures were approved by the University of Maryland Baltimore Institutional Animal Care and Use Committee.

## Data Availability

The data that support the findings of this study are available from the corresponding author upon reasonable request.
